# Permanent Maxillary and Mandibular Central Incisor Width as Predictor of Permanent Maxillary Canine Width in a Kurdish Population: A Pilot Study

**DOI:** 10.3390/children7080092

**Published:** 2020-08-06

**Authors:** Fadil Abdullah Kareem

**Affiliations:** Orthodontics and Preventive Dentistry, Department of Pedodontics, College of Dentistry, University of Sulaimani, Sulaymaniyah, 46001, Iraq; fadil.kareem@univsul.edu.iq; Tel.: +(964)-770-1541370

**Keywords:** prediction formula, mesio-distal tooth width, permanent maxillary canine, permanent maxillary and mandibular central incisors, Kurdish

## Abstract

Background: Estimation of the mesio-distal width of permanent maxillary canines (PMCs) is a critical part of mixed dentition space analysis. The aim of this pilot study is to find a specific prediction equation for the estimation of the mesio-distal width of PMCs depending on the width of permanent maxillary and mandibular central incisors (PMMCIs) in a Kurdish population. Methods: A hundred study casts were collected. The mesio-distal widths of the PMMCIs and PMCs were measured by digital caliper. Linear regression tests were applied to find the prediction equation using the sum width of PMMCIs as predictors. Results: The mean age of the subjects was 17.2 ± 2.39 years old. Statistically significant differences in the mesio-distal widths of PMMCIs and PMCs were found between males and females (*p* = 0.0001). Furthermore, statistically significant correlations were identified between the widths of PMMCIs and PMCs in both males (r = 0.633, *p* = 0.0001) and females (r = 0.717, *p* = 0.0001). Likewise, the mesio-distal width of PMMCIs was found to be a significant predictor of the width of PMCs in both males (R2 = 0.403, *p* = 0.0001) and females (R2 = 5.14, *p* = 0.0001). Conclusion: For the first time, regression equations were developed for a Kurdish population and can be useful as a part of a mixed dentition space analysis in Kurdish children.

## 1. Introduction

Predicting the development of occlusion in children and adolescents is of paramount importance for dentists providing dental care for them [1]. Orthodontists and pedodontists are often required to provide accurate diagnosis for any future malocclusion and problems associated with it. Mixed dentition space analysis (MDSA) is critical in early diagnosis of malocclusion as many malocclusions may emerge in the age range of 6 to 12 years; thus, a proper intervention at this time would be helpful to prevent or minimize the severity of the malocclusion [2]. Mixed dentition space analysis assesses the need for preventive and interceptive measures, which are important to prevent a potential dental malalignment from progressing into a more severe malocclusion [3]. The preventive or interceptive measures that can be used based on MDSA could be guidance of eruption such as operculectomy, serial extraction, proximal stripping, regaining space or just continuous checkup [4,5,6].

Three techniques have been used to estimate the mesio-distal width of unerupted premolars and canine crowns, including measurements from erupted teeth, radiographs and a combination of measurements from erupted teeth and radiographs of unerupted teeth [7]. From a practical point of view, these analyses have to be simple, quick, reliable, not requiring special tools, and must be specific for each arch [3]. Taking measurements from radiographs requires time, specific equipment, and is less practical as undistorted radiographic images are required. Moreover, the radiation burden is always unjustifiable [8].

Tables from Moyers for MDSA [9] and equations from Tanaka and Johnston [10] are the oldest methods relying on a correlation of tooth size. These methods use measurements of one tooth or a group of teeth to predict the size of the other teeth accurately in the oral cavity. According to the Moyers analysis, the selection of mesio-distal width of the permanent mandibular incisors (central and lateral incisors) to estimate the width of the permanent maxillary canines (PMCs) and premolars in both arches is based on the early eruption of permanent mandibular incisors in the mixed dentition and the ability to measure them accurately [8].

Several studies have been conducted using Moyers analysis in different populations; however, it has been reported that the prediction methods are not accurate in other populations [4,11,12]. This could be on account of tooth size varying between different ethnic groups, genders [13,14,15], as well as other factors such as genetic and environmental factors [16,17]. Similarly, due to the aforementioned factors, Moyers analysis and Tanaka and Johnston prediction methods have been found to be inaccurate when applied to a Kurdish population [18].

The PMCs are considered as a corner stone of occlusion and play a key role in smile design [19]. Furthermore, PMCs are the last teeth to erupt in the sequence of normal eruption of the permanent maxillary teeth [20]. The length of PMCs’ path of eruption, the magnitude and timing of eruption are very difficult to determine [21]. On the other hand, the prevalence of malposed PMCs is very high in the Iraqi population. For example, in a Kurdish population, the prevalence of abnormally positioned PMCs among subjects aged 12–22 years was 5.35% and the most common problems included malposition (50%), followed by canine rotation, displacement, impaction and transposition [22]. Moreover, in a sample of the Iraqi population, the prevalence was found to be higher (9.72%) [23].

It is important to acknowledge that no study has yet been carried out among a Kurdish population to determine the mesio-distal width of unerupted PMCs. Being able to anticipate the future impaction or malocclusion of PMCs would be of great value in terms of potentially leading to early intervention to prevent or reduce the impact of the associated problems. In general, the quality of the prediction method based on regression analysis depends on the selected independent variables. In the United States, the sum width of mandibular incisors has been preferred as the independent variable [9,24]. In contrast, the European schools prefer the sum width of maxillary incisors as the independent variable [25]. In this study, the sum width of permanent maxillary and mandibular central incisors (PMMCIs) were examined as the independent variable that represents the combination of both schools. Accordingly, the aim of the present pilot study is to find a specific prediction equation for the estimation of mesio-distal width of PMCs that can be useful as a part of MDSA and depends on the sum width of PMMCIs in a sample of the Kurdish population. There was a null hypothesis of no association of width of PMMCIs with width of PMCs.

## 2. Materials and Methods

### 2.1. Study Design

This cross-sectional study was based on measuring the dimensions of selected teeth after examining the dental files of study casts of Kurdish subjects who had orthodontic treatment in private dental clinics in the Kurdistan region of Iraq from December 2017 to April 2019. Ethical approval for the study was obtained from the ethical committee of the Medical Colleges/University of Sulaimani (ethical approval number: 395) in accordance with the Helsinki declaration.

### 2.2. Study Sample

A total of 100 study casts were randomly selected for this analysis, the age of the included subjects ranging from 13 to 24 years old. The inclusion criteria were: presence of all permanent teeth in both jaws, no interproximal caries or restoration, no previous history of orthodontic treatment from dental file of study subjects, no congenital anomalies and no attrition in occlusal and interproximal surfaces.

### 2.3. Measurements and Study Outcomes

**Figure 1:**
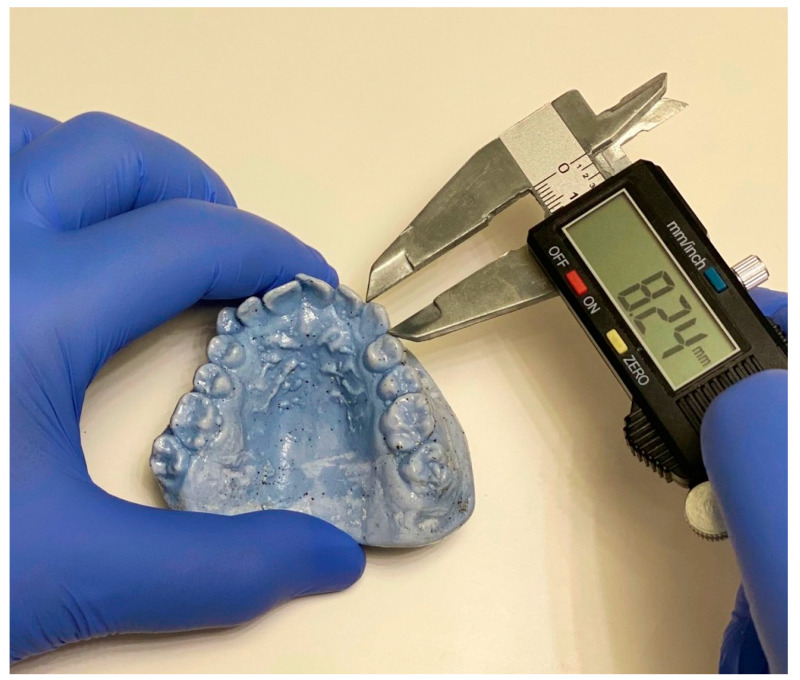
Digital caliper used in the study.

The largest mesiodistal widths of the PMMCIs and PMCs were measured using a digital caliper (Mitutoyo, Tokyo, Japan) with 0.001 mm accuracy for each study cast (Figure 1). The widths of both the right and left sides of the PMMCIs were measured, from which the width of the PMCs was determined. Intra-examiner calibration was performed on 20 study casts not included in the study, with a one-week interval between the calibrations (correlation test = 0.98).

### 2.4. Statistical Analysis

The normality test (Shapiro–Wilk test) for continuous data was performed and the data were then subjected to an appropriate test. Independent *t* test was used to compare age, sum width of PMMCIs and PMCs between males and females as these data were shown to be parametric. Pearson correlation was used to check the association between the widths of PMCs and PMMCIs. A linear regression test was applied to attain the equation for prediction of mesio-distal width of PMCs depending on the sum of mesio-distal width of PMMCIs. Statistical significance was defined as *p* ≤ 0.05 and all calculations were conducted using GraphPad Prism (version 8.4.0) software. Advice on data analysis was provided by an expert statistician.

## 3. Results

Study casts of 100 subjects (50 male and 50 female) with mean age 17.2 ± 2.39 (ranging from 13–24 years old) were included in the study. The sum widths of the PMMCIs and PMCs were 31.5 ± 1.9 mm and 16.1 ± 1.02 mm, respectively (Table 1). No statistically significant differences in mean age were found between males and females. However, a statistically significant difference in the mesio-distal widths of PMMCIs and PMCs was identified between males and females (*p* = 0.0001, Table 1).

There was a statistically significant association between the widths of PMCs and PMMCIs. The strength of association was higher in females (r = 0.717, *p* = 0.0001) than males (r = 0.633, *p* = 0.0001) (Table 2). Regression analysis showed that the width of PMCs can be statistically significantly predicted using the sum mesio-distal width of PMMCIs. Similarly, the level of prediction was higher in females (R^2^ = 0.514, *p* = 0.0001) than males (R^2^ = 0.403, *p* = 0.0001) (Table 3). Moreover, the Standard Errors of Estimate (SEE) in the equations for males and females were 0.059 and 0.049, respectively.

**Figure 2:**
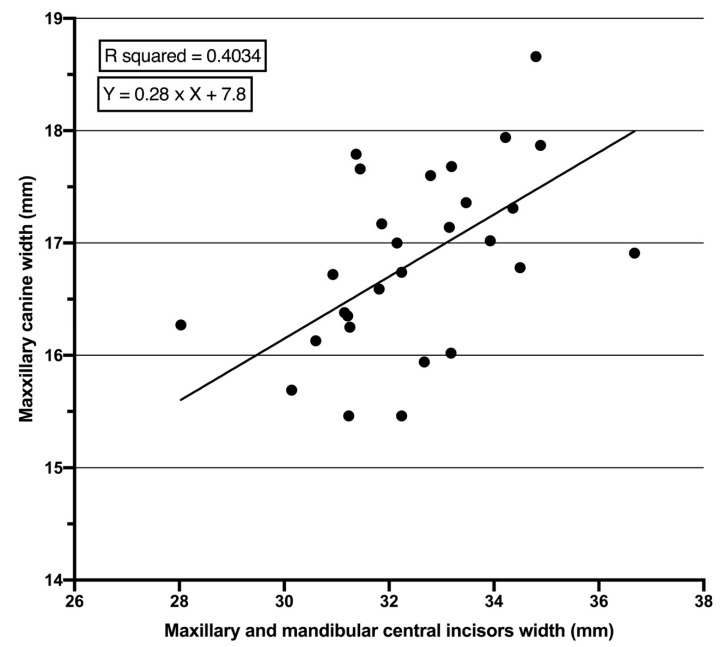
Linear regression line between mesio-distal widths of permanent maxillary and mandibular central incisors and mesio-distal widths of permanent maxillary canines in males.

**Figure 3:**
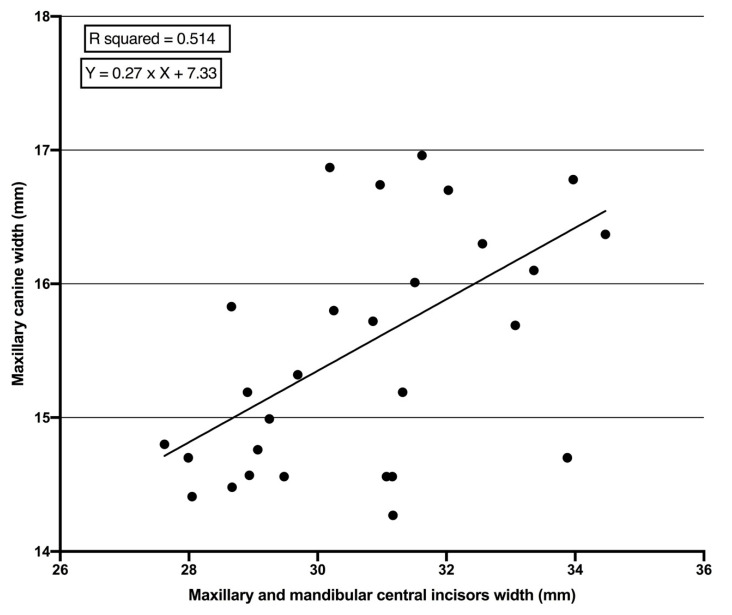
Linear regression line between mesio-distal widths of permanent maxillary and mandibular central incisors and mesio-distal widths of permanent maxillary canines in females.

The equation was shown as Y = a × X + b, where Y stands for the mesio-distal width of PMCs, the constant “a” is the slope of regression, “b” is the Y intercept and X is the sum of the mesio-distal widths of PMMCIs. The prediction equation was Y = 0.28 × X + 7.8 for males (Figure 2) and Y = 0.27 × X + 7.33 for females (Figure 3).

## 4. Discussion

Prediction of the mesio-distal width of unerupted teeth during MDSA has to be accurate as the treatment plan mostly relies upon it [26]. Ethnic background has been shown to have an impact on tooth size and consequently variation in the prediction method for MDSA [13,14,15]. The linear regression equations were developed using the sum width of PMMCIs as the predictor for the sum of the width of PMCs, which can be useful as a part of the MDSA for the Kurdish population. The underestimation or overestimation of the crown width of unerupted canines can affect the treatment plan. For example, in the case of underestimation, canines that are larger and of greater width can be expected; thus, more space will be necessary than planned, which might lead to late or unplanned extraction of permanent teeth. Whereas, in the case of overestimation, smaller canines than planned can be expected; consequently, unnecessary extraction of teeth might have taken place at the beginning of orthodontic treatment [25].

There is no universal prediction method across all ethnicities to predict the size of unerupted teeth as teeth size varies across different ethnic groups [4,11,12]. The cause for such variation is not yet clear; however, genetics, environment and nutrition all play important roles [27]. Consequently, having a reliable method of predicting tooth size for each racial group is highly recommended [11]. The rationale behind this study to predict the width of PMCs as a part of MDSA is as follows: first, the Moyers method to predict the width of PMCs and permanent maxillary premolars has been shown to be inaccurate in a Kurdish population and this could be related to genetic, environmental and nutritional factors, as Moyers’ study was conducted on the white American ethnic group [18]; second, the European schools prefer maxillary central incisors as predictors [25]. Selecting both maxillary and mandibular incisors has several advantages, for example, they erupt early in mixed dentition, are easily measured with little variation in size and located in the middle of the space treatment problem [9]. Thus, this pilot study aimed to use the mesio-distal width of PMMCIs to predict the mesio-distal width of PMCs as a part of MDSA in a Kurdish population. The age range of the subjects included in the study is from 13–24 years old because the PMCs usually erupt at the age of 11–12 years old (if not erupted at 13 years old, it is considered as impacted tooth) and to exclude the effect of interdental attrition, which starts at age of 25 years old (thus, those up to 24 years old are included) and apparently this attrition affects the width of PMMCIs and PMCs [28].

In addition to racial differences in tooth size between the Kurdish and Moyers populations [18], there were statistically significant differences in the mesio-distal widths of PMMCI and PMCs between males and females (Table 1). The mesio-distal width of examined teeth (PMMCIs and PMCs) was shown to be higher in males than females, which is in line with other studies [3,4,11,12]. This difference has been reported to be associated with X-linked inheritance, thus, having 2 X chromosomes in females enhances the accuracy of size prediction compared to males, who have one X chromosome [27,29]. Whatever the reason for the gender teeth size difference, it emphasizes the importance of developing different prediction equations for males and females in each ethnic group, to enable more precise teeth width prediction during MDSA.

The correlation coefficients of PMMCIs and PMCs were shown to be statistically significant in both males and females (Table 2). The Pearson correlation was revealed to be higher in females than males; again, this could be related to the X-linked inheritance in females [27,29]. This study’s findings on correlation coefficient values are in agreement with other studies such as Lee-Chan et al. [7] (0.66), Tanaka and Johnston [10] (0.65), Ballard and Wylie [24] (0.64), and Hixon and Oldfather [30] (0.69). These relatively consistent associations (0.6–0.7) mean that about 60–70% of genes that determine tooth size are shared between PMCs and PMMICs [10,27].

The high level of Pearson correlation between the sum of PMMCIs and PMCs (Table 2) means that the width of unerupted PMCs can be measured based on the width of PMMCIs. The coefficients of determination (R^2^), which represent the accuracy of the regression equation, were statistically significant for both females and males (Table 3). The R^2^ means that 51.4% and 40.3% of the total variances in PMCs’ width can be accounted for by knowing the sum of PMMIC width in females and males, respectively. The R^2^ values produced in this study are comparable with results from Saudi Arabian [31] and Senegalese samples [3], but smaller than in Hong Kong samples [32] and higher than in Pakistani samples [12]. The SEE values represent the error in the prediction equations, i.e., the lower the SEE, the more accurate the prediction equation. In the current study, the SEE values are below 0.1 mm for both genders, which is comparable to values in a Turkish population [33], but lower than in Indian [4], White Caucasian [9,10] and Thai [34] populations. The smaller SEE values in the present study mean that the prediction equations used to determine the sum of the PMCs’ width based on the sum of PMMCIs’ width are more accurate.

Several studies show that racial differences do affect the prediction equation [4,11,12]; accordingly, developing individual prediction equations for each ethnic background rather than relying on popular prediction methods is highly recommended. The proposed prediction equations for Kurdish subjects (male and female) can be used in a Kurdish population to determine the mesio-distal width of unerupted PMCs when the PMMCIs are completely erupted, which can be useful as a part of MDSA (Figure 2 and Figure 3). The study has some limitations, such as small sample size and no other ethnic groups in the examined area included, and, because of the small sample size, the validation could not be performed. Therefore, further studies in the Kurdistan region/Iraq are required that are based on larger sample sizes and more ethnic groups (Turkish and Assyrian). Moreover, validation of this study’s results within the same population is necessary to confirm the precision and reliability of these regression equations.

## 5. Conclusions

This pilot study found that males had statistically significant higher mesio-distal widths of PMMCIs and PMCs compared to females. The correlation and determination coefficients of PMMCIs and PMCs were shown to be statistically significant in both males and females. For the first time within a Kurdish population, linear regression equations were developed using the sum width of PMMCIs as predictors for the sum of the width of PMCs, which can be useful as a part of MDSA.

## Figures and Tables

**Table 1:** Comparison in age and sum of widths of examined teeth between males and females (*n* = 100).

Variables	Minimum	Maximum	Mean, SD	Male	Female	Male vs. Female *
Age (years)	14	24	17.2 ± 2.39	17.5 ± 2.66	16.9 ± 2.06	0.21
Sum of PMMCI widths (mm)	27.62	36.68	31.5 ± 1.9	32.4 ± 1.6	30.6 ± 1.9	0.0001
Sum of PMC widths (mm)	14.27	18.66	16.1 ± 1.02	16.8 ± 0.75	15.5 ± 0.8	0.0001

* independent *t* test.

**Table 2:** Pearson correlation coefficient between permanent maxillary canines and permanent maxillary and mandibular central incisors.

		Mesio-Distal Width of PMMCIs	
		Male	Female
Mesio-distal width of PMCs	Pearson Correlation	0.633	0.717
Sig. (2-tailed)	0.0001	0.0001

**Table 3:** Regression analysis for prediction of the sum width of permanent maxillary canines from the sum of widths of permanent maxillary and mandibular central incisors.

Teeth Width	Width of Permanent Maxillary Canines						
	Gender	R	R Square	Std. Errors of the Estimate	95% CI	*t*	*p* Value
Permanent maxillary and mandibular central incisors	Male	0.633	0.403	0.059	0.167–0.386	5.07	0.0001
	Female	0.717	0.514	0.049	0.169–0.366	5.46	0.0001
